# Supplementary material for: Long-Term Response to Tyrosine Kinase Inhibitors for Metastatic Renal Cell Carcinoma
Source: Biomedicines. 2022 Sep 30;10(10):2444. doi: 10.3390/biomedicines10102444 (PMC9599295; doi:10.3390/biomedicines10102444)
Supplement: Supplementary file 1 [file biomedicines-10-02444-s001.zip › biomedicines-1912491-supplementary.pdf]

Table S1: Best response, according to duration of response to TKI.

	RR, n (%)	*p*	DCR, n (%)	*p*
**All patients**	124 (37.1)		274 (81.8)	
PFS≥36 months	36 (54.5)	<0.01	66 (100)	<0.01
PFS<36 months	88 (32.7)		208 (77.3)	
**Favoravle Risk**	54 (46.5)		106 (91.4)	
PFS≥36 months	19 (61.3)	0.05	31 (100)	0.05
PFS<36 months	35 (41.2)		75 (88.2)	
**Intermediate-poor risk**	70 (32)		168 (76.7)	
PFS≥36 months	17 (48.6)	0.03	35 (100)	<0.01
PFS<36 months	53 (28.8)		133 (72.3)	

RR: response rate; DCR: disease control rate; p: p value

Table S2: Progression free survival according to duration of response to prior TKI.

Patients	mPFS months (95% CI)	Hazard Ratio (95%CI)	*p*
All patients	13 (11-15)		
PFS≥36 months	70 (58-86)	0.00 (0)	<0.01
PFS<36 months	10 (8-12)		
**Favorable risk**			
All patients	21 (15-31)		
PFS≥36 months	72 (52-86)	0.00 (0)	<0.01
PFS<36 months	13 (12-18)		
**Intermediate-poor risk**			
All patients	11 (9-12)		
PFS≥36 months	67 (55-100)	0.00 (0)	<0.01
PFS<36 months	8 (7-10)		

PFS: progression free survival; CI: confidence interval; NR: not reached; m: median.

Table S3: Overall survival according to duration of response to prior TKI.

**Patients**	**mOS months (95% CI)**	**Hazard Ratio (95%CI)**	***p***
All patients	48 (39-60)		
PFS≥36 months	106 (93-NR)	0.20 (0.12-0.32)	<0.01
PFS<36 months	33 (24-39)		
**Favorable risk**			
All patients	75 (63-105)		
PFS≥36 months	105 (70-NR)	0.30 (0.14-0.62)	<0.01
PFS<36 months	66 (40-75)		
**Intermediate-poor risk**			
All patients	33 (24-43)		
PFS≥36 months	112 (93-NR)	0.17 (0.09-0.32)	<0.01
PFS<36 months	23 (20-29)		

OS: overall survival; CI: confidence interval; NR: not reached; m: median.
